# Exploring the challenges of a pilot dental services payment initiative: a qualitative policy analysis at an Iranian dental school

**DOI:** 10.1186/s12903-026-08767-9

**Published:** 2026-06-29

**Authors:** Iman Rajaei, Seyed Hadi Hosseini, Saleheh Khorasani, Azadeh Babaei

**Affiliations:** 1 Student Research Committee, Alborz University of Medical Sciences, Karaj, Iran; 2 Department of Healthcare Services Management, School of Public Health, Alborz University of Medical Sciences, Karaj, Iran; 3 Social Determinants of Health Research Center, Alborz University of Medical Sciences, Karaj, Iran; 4 Community Oral Health Department, School of Dentistry, Alborz University of Medical Sciences, Karaj, Iran

**Keywords:** Payment initiative, Dental Services, Policy Analysis, Qualitative Study, Iran

## Abstract

**Background:**

Payment initiative in dental services is a key policy strategy to enhance access, affordability, and patient satisfaction. The Ghasedak project, a six-month pilot initiative at a university dental school in Iran, aimed to test a new payment model. This study analyzed the reasons for its suspension to draw lessons for future initiatives.

**Methods:**

A qualitative study was conducted using Walt and Gilson’s policy triangle framework. Data were collected in 2024 through 19 semi-structured interviews with project stakeholders (founders, managers, providers, and philanthropists) and 28 patients, selected via purposive sampling at Alborz University of Medical Sciences, Karaj. Data were analyzed using the Elo and Kyngäs inductive content analysis approach in MAXQDA, consisting of preparation, organization, and reporting phases.

**Results:**

From 145 initial codes, 13 themes and 61 subthemes were identified. Mapped to the policy triangle, barriers included: Context (management instability, lack of institutionalized inter-sectoral cooperation, insufficient upstream support); Content (lack of an independent and transparent structure, unsustainable funding, non-adherence to social work principles, and lack of continuity in service delivery); Process (administrative bureaucracy, decreased financial transparency, lack of service support); Actors (low patient satisfaction stemming from mistrust, lack of motivation of stakeholders, and unrealistic patient expectations).

**Conclusions:**

Structural, managerial, and operational deficiencies undermined the pilot’s sustainability and impact. Strengthening future payment initiatives requires secure financing, coherent management, streamlined implementation, and genuine stakeholder engagement. These findings provide actionable insights for policymakers seeking to design more resilient and effective dental payment initiative.

**Supplementary Information:**

The online version contains supplementary material available at https://doi.org/10.1186/s12903-026-08767-9.

## Background

Payment reform projects in dental service delivery, one of the relatively expensive areas of health services, are considered key tools for improving oral health outcomes, patient access, and satisfaction in dentistry [1]. The World Health Organization (WHO) underscores the importance of financial protection in achieving universal health coverage (UHC) [2], particularly in the Middle East, where high out-of-pocket dental expenditures are a major contributor to catastrophic health costs [3]. Elevated out-of-pocket payments have been linked to reduced treatment-seeking behavior, widening health inequalities, and increasing catastrophic dental expenses, especially among lower-income populations [4].

Recent literature highlights a paradigm shift from fee-for-service to value-based and blended capitation models, which have proven effective in enhancing access and incentivizing preventive care over volume-driven treatments [5–7]. To address the related payment challenges, a range of models has been explored. Fee-for-service systems, which remain common in dental practice, often incentivize overtreatment, whereas capitation models can encourage undertreatment and compromise care quality [8, 9].

**Fig. 1:**
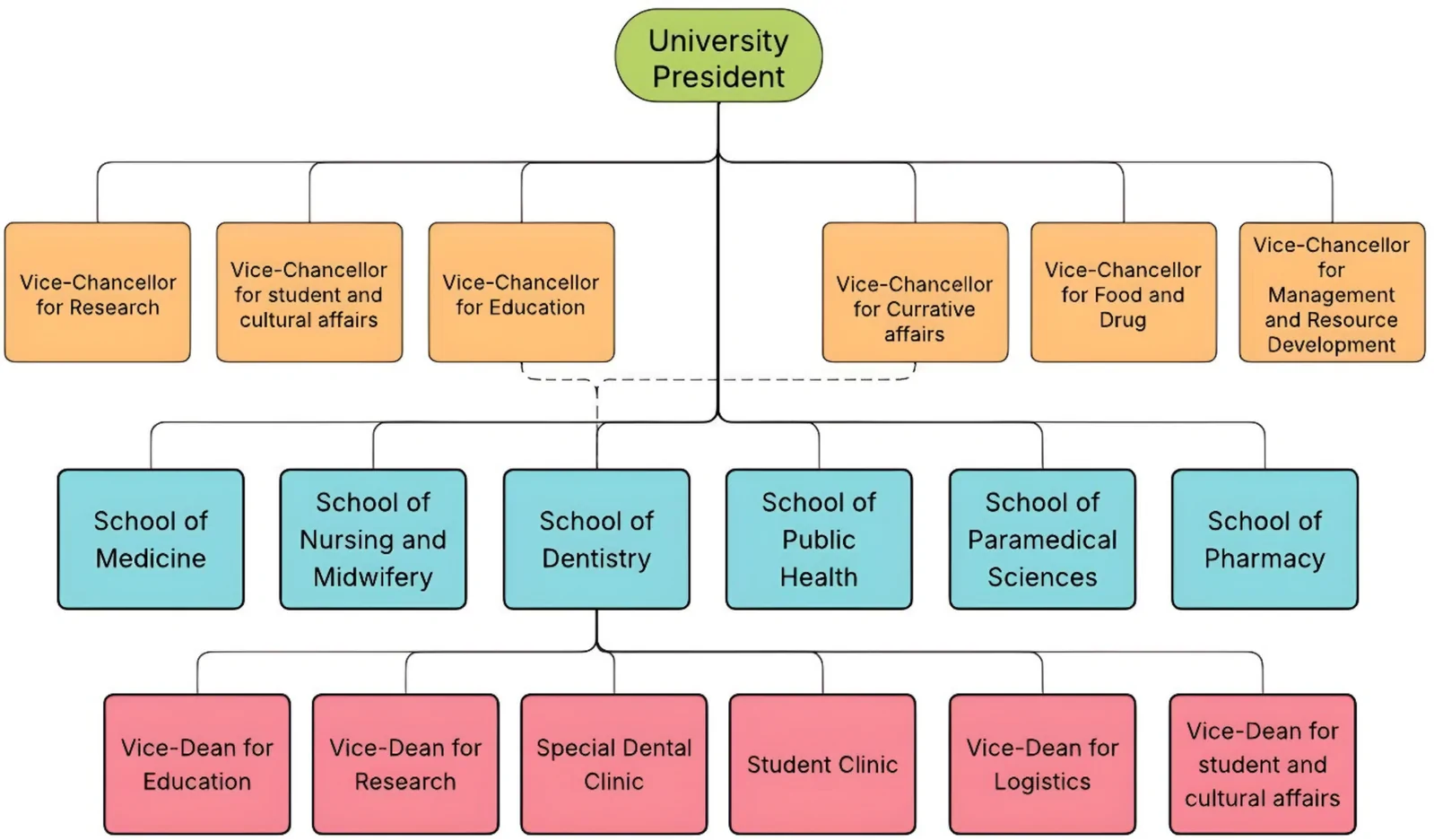
The position of dental clinics within the organizational structure of Iranian medical sciences and health services universities: the case of Alborz University of Medical Sciences

In Iran, since the integration of medical education and healthcare service delivery in 1985, dental faculties under the Ministry of Health and Medical Education (MoHME) supervision have operated within a dual mandate to improve both educational quality and service provision [10] (Fig. 1). This integration has enabled patients to receive subsidized dental care delivered by students under faculty supervision through a co-payment system, where the government covers part of the cost and patients pay a significant out-of-pocket share. Despite these subsidies, the payment structure has limited patient utilization of dental services at academic institutions, raising concerns about equity, access, quality, and the provision of preventive oral health services [11–13]. And also, this co-payment system has shifted government budget allocations toward service delivery at the expense of resources for education and research [14].

Furthermore, approximately 95% of the population in Iran is covered by various health insurance schemes [15]. One of these health insurance schemes, is the Imam Khomeini Relief Foundation (IKRF), which specifically supports low-income groups. However, Iran’s dental insurance varies in diversity of services and financial limits, leading to access inequalities, especially for low-income groups [16, 17]. These inequalities highlight the need for payment initiatives to improve coverage and equity [18]. Innovative projects, like strategic purchasing or subsidies, could address these gaps, though implementation challenges persist, motivating further policy development [16–18].

One of innovative approaches to reduce oral health burdens, is integration of insurance funds and the dentistry faculties of MoHME, to reduce patients’ financial burdens and alleviate pressures, potentially enabling the reallocation of government resources to education and research [19].

**Fig. 2:**
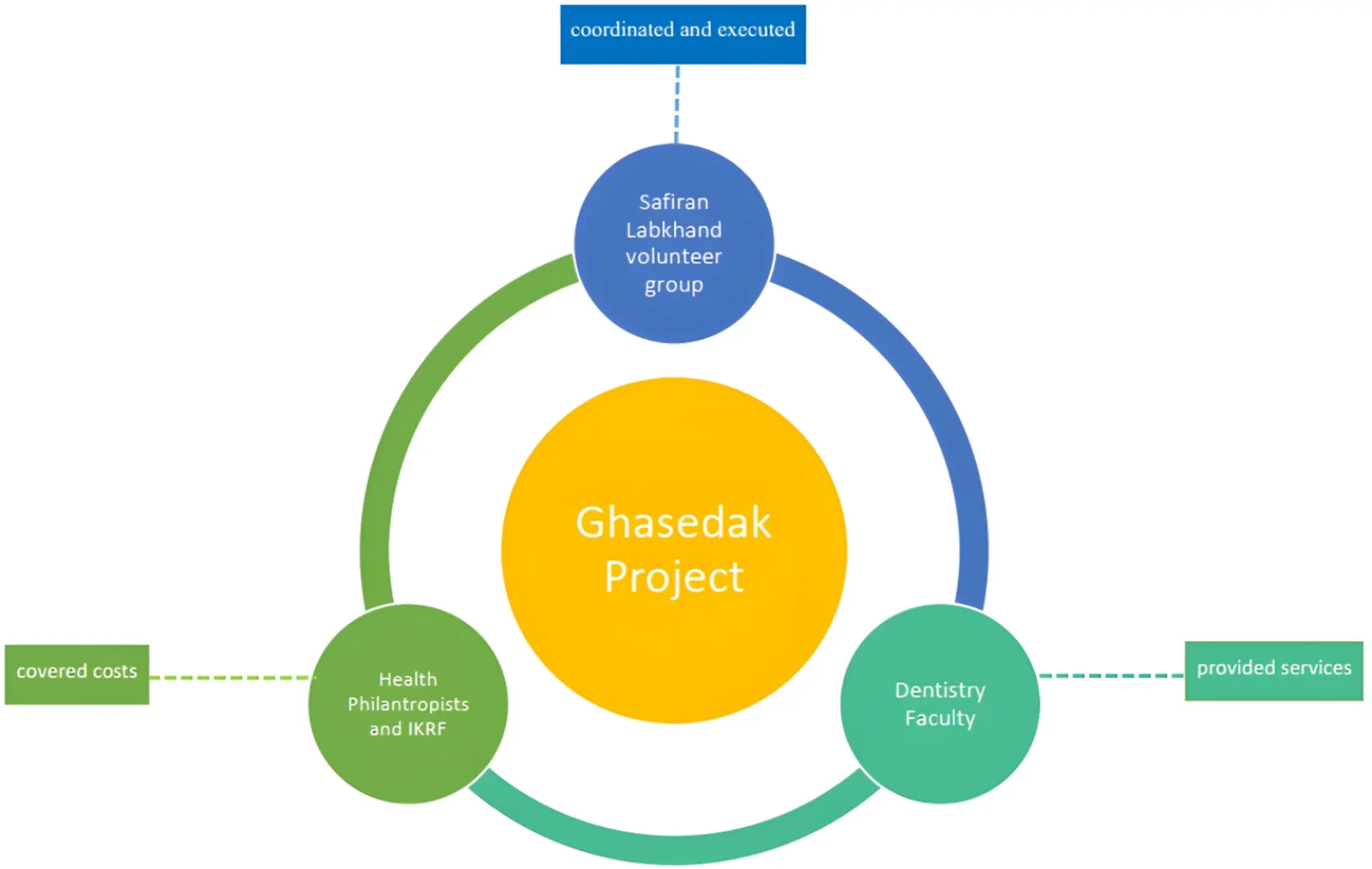
“Ghasedak” pilot project: A payment model for dental services delivery in Alborz Medical University Dental Clinic

To explore a more sustainable payment model, the “Ghasedak” pilot project (Fig. 2) was launched at the Dentistry School of Alborz University of Medical Sciences (ABZUMS) from February 2023 to July 2023 (“Project” refers to “Ghasedak Project” hereafter). This initiative was implemented over six months through a consortium comprising the IKRF insurance fund and health charities as financial providers, the dental faculty as service provider, and the “Safiran Labkhand” volunteer group as implementer and facilitator. The Project offered free dental treatments to patients, aiming to test the feasibility of integrating insurance and subsidy mechanisms in an academic setting to enhance oral health outcomes, access, equity, and equality.

Through the efforts of the volunteer group, an agreement was established between the faculty of dentistry and the IKRF. By this contract, philanthropists and the IKRF covered the costs of dental consumables, while the faculty committed to providing free dental services to referred patients. The volunteer group was tasked with coordinating and executing related activities.

Following the project’s launch, free dental services were provided to patients referred by the IKRF every Wednesday (the last working day of the week), with material costs borne by the IKRF and health philanthropists. On other weekdays, services continued as usual for all patients. Despite all expected advantages, this project faced challenges!

To recognize a critical gap in the literature, this study utilizes a qualitative design guided by Walt and Gilson’s policy triangle to move beyond simple outcome measurement and holistically analyze the specific barriers that inhibit the sustainability of inter-organizational public sector dental payment initiatives in developing contexts. Policy analysis of such projects, by identifying their weaknesses, can generate practical recommendations to improve similar interventions and support potential nationwide scaling. Therefore, this study aimed to analyze the challenges and reasons associated with the suspension of the pilot payment initiative in the dental school of a medical university.

## Methods

### Study design

This study employed a qualitative policy case study design guided by Walt and Gilson’s policy triangle framework, which analyzes health policy through four dimensions: context, content, process, and actors [20]. This design enabled a retrospective and in-depth analysis of the project to identify the reasons for its implementation challenges and to inform future interventions.

### Study setting and participants

The study was conducted at the school of dentistry affiliated with Alborz University of Medical Sciences, located in Karaj, the capital of Alborz Province, Iran. Karaj is a rapidly urbanizing city near Tehran, representing a socioeconomically diverse population and growing healthcare needs. The university, founded in 2010, serves as a regional academic and service hub with an emphasis on educational innovation and community-based healthcare. The school of dentistry, as one of its core faculties, combines dental education with patient service delivery under the national integration of health and medical education. At the time of the study, the dentistry school had about 50 board-certified faculty members, trained about 500 students, and its dental clinic admitted approximately 3,000 patients per year.

Key and informed stakeholders were selected using purposive and snowball sampling strategies. Initial founders and project initiators were identified first; they subsequently referred us to operational managers and volunteers with specific knowledge of the project’s financial and administrative processes. These informants included: Founders and initiators of the project, Executive and operational managers within the faculty and affiliated organizations, Dental care providers (dental students) and clinical supervisors, Representatives of the Imam Khomeini Relief Foundation (IKRF), Volunteers from the “Safiran Labkhand” group, and Philanthropic health benefactors.

Selection criteria included having first-hand knowledge or experience in the planning, implementation, or supervision of the project, and willingness to participate in the study. However, incomplete and irrelevant interviews were excluded. Patients who had received services through the project were identified from the faculty’s clinical records. Patients were purposively sampled [21] to ensure demographic diversity (age and sex), with inclusion based on verified service use and informed consent obtained via telephone. Exclusion criteria included inability to recall the service, refusal to participate, or failure to respond to multiple attempts at contact.

### Data collection

Data were collected in two stages [22, 23]. In the first stage, 19 semi-structured interviews were conducted with the identified key informants, and in the second stage, 28 semi-structured interviews were held with patients to triangulate user perspectives.

To ensure in-depth engagement with the study context, interviews were conducted by a member of the research team who was a senior dental student at the time and a former member of the “Safiran Labkhand” volunteer group. All interviews were carried out under the supervision of the corresponding author. Data collection took place between November 2024 and April 2025 and was conducted face-to-face (11 key informants), by telephone (7 key informants and 28 patients), or via Google Meet (1 key informant). Face-to-face interviews were conducted in the participants’ offices. Each session lasted approximately 20–50 min and followed a semi-structured guide refined through pilot testing. Interviews with patients were conducted by telephone. Patients’ contact information, available in the dental clinic’s records, was used to reach them and arrange these interviews. In telephone interviews and the Google Meet interview, initially a call was made to inform about the interview and obtain informed consent. General aspects of the interview were provided for review, then the main interview was conducted, lasting approximately 15–30 min. To mitigate potential insider bias, the interviewer strictly adhered to the semi-structured guide. All interviews were audio-recorded with participants’ informed consent.

Interview questions explored: Project experiences, perceived strengths and weaknesses, factors contributing to project challenges, categorized by the four elements of the Walt and Gilson framework, and Suggestions for future improvements. Data collection continued until theoretical saturation was reached; this was operationalized as the point where three consecutive interviews yielded no new codes or sub-themes.

### Data analysis

Data analysis was conducted concurrently with data collection using inductive qualitative content analysis, following the methodology proposed by Elo and Kyngäs [24], supported by MAXQDA 24 software. This approach was selected to allow systematic abstraction of meaning from textual data while remaining grounded in participants’ perspectives. The analysis followed the three sequential phases outlined by Elo and Kyngäs: (1) Preparation, during which the unit of analysis was defined, and all transcripts and documents were read repeatedly to achieve immersion; (2) Organization, which involved open coding and the iterative grouping of codes into 61 sub-themes and 13 higher-order themes; and (3) Reporting, where themes were further abstracted and integrated into the final analytical framework.

Coding was performed inductively, guided by the research questions and emerging patterns in the data, with attention to similarities, differences, and contradictions across data sources. After finalizing the inductive themes, three authors collaboratively mapped each theme onto the four dimensions of the Walt and Gilson Policy Triangle through a structured focus group discussion. Any discrepancies were resolved through consensus-based deliberation under the supervision of the corresponding author.

### Rigor and trustworthiness

To ensure rigor and trustworthiness, several established qualitative strategies were applied. Prolonged engagement was achieved through continuous interaction with policy documents and key informants throughout data collection and analysis. Alongside the interviews, we reviewed gray literature, including official contracts, meeting minutes, and administrative reports generated both prior to and during the project’s implementation. Two authors independently reviewed these documents using content analysis guided by the research question to cross-verify the identified codes, and ensure comprehensive coverage of the data collected through interviews. Member checking involved sharing preliminary findings with selected participants to confirm the accuracy of interpretations. Peer debriefing was conducted through regular discussions with independent qualitative and health policy experts to review coding and analytic decisions. In addition, a comprehensive audit trail documenting methodological and analytic processes was maintained, enhancing the study’s credibility, dependability, and transferability in line with qualitative research standards [22, 23]. Furthermore, a dual-verification process was implemented. Immediately following each interview, the primary researcher transcribed the audio verbatim. To verify accuracy and minimize bias, a second independent researcher re-transcribed and coded the data separately. Both researchers recorded their findings in separate codebooks using MAXQDA 24. A cross-check was performed between the authors after each interview. Any discrepancies in coding or interpretation were resolved through a focus group discussion involving a third senior researcher to reach a consensus. To preserve data integrity, no codes were manipulated or merged until the final reporting phase. Finally, the translation of concepts from Persian to English was validated by three authors: a PhD in Healthcare Services Management, a PhD in Community Oral Health, and an MPH in Health Policy, to ensure conceptual equivalence and semantic accuracy.

## Results

A total of 47 participants were interviewed for this study. Of these, 13 (27%) were key stakeholders, 6 (13%) were informed stakeholders (dental students), and 28 (60%) were patients who had received services. The demographic characteristics of the key (No.1 to No.13) and informed (No.14 to No.19) stakeholders are presented in Table 1. To protect participant confidentiality, all demographic and professional characteristics are presented in aggregated and generalized categories. Potentially identifying combinations of roles and descriptors is avoided, particularly for participants occupying unique or senior positions. The patient group included 26 women and 2 men, with a mean age of 46 years.

**Table 1:** Demographic characteristics of key and informed stakeholders in the study (at the time of interview)

Participant number	Gender	Position in the project	Interview type
1	Female	Faculty administrative staff	face-to-face
2	Female	Faculty administrative staff	face-to-face
3	Female	Faculty administrative manager	face-to-face
4	Female	Faculty administrative manager	face-to-face
5	Male	Faculty administrative manager	face-to-face
6	Male	Safiran Labkhand volunteer group stakeholder	face-to-face
7	Male	Safiran Labkhand volunteer group stakeholder	Google Meet
8	Female	Safiran Labkhand volunteer group stakeholder	face-to-face
9	Male	Imam Khomeini Relief Foundation administrative manager	face-to-face
10	Male	Health philanthropist	telephone
11	Male	Imam Khomeini Relief Foundation administrative staff	face-to-face
12	Male	Imam Khomeini Relief Foundation administrative staff	face-to-face
13	Male	Imam Khomeini Relief Foundation administrative staff	face-to-face
14	Female	4th year dentistry student	telephone
15	Female	6th year dentistry student	telephone
16	Male	5th year dentistry student	telephone
17	Male	6th year dentistry student	telephone
18	Female	5th year dentistry student	telephone
19	Male	6th year dentistry student	telephone

### Content analysis

Following the interviews and subsequent data analysis, a total of 145 open codes were extracted from the qualitative data. These open codes were systematically reviewed, compared, and grouped using an inductive content analysis approach. As a result, they were organized into 61 sub-themes and 13 overarching themes. Table 2 presents the detailed classification of these themes and sub-themes, illustrating the final conceptual structure derived from the coding process.

**Table 2:** Themes and sub-themes associated with them

Theme	Sub-themes
Lack of an Independent and Transparent Structure	Undefined Quality Management System
	Delegating Assigned Tasks to Others
	Lack of a Suitable Implementation Model
	Lack of Long-Term Planning
	Lack of Meritocracy in Managerial Appointments
	Lack of Precise and Correct Role Definition
	Irregular Meetings
	Decrease in Organizational Discipline
	Lack of Coordination in Consumable Supply
	Lack of an Independent Accounting System
	Lack of an Independent and Coherent Legal Basis
	Lack of an Independent Consumption Management System
Management Instability	Short Managerial Tenure of the Dean of the Faculty of Dentistry (Service Provider Unit)
	Short Managerial Tenure of the Head of the IKRF (Project Coordinator)
Low Patients Satisfaction stemming from Mistrust	Patient Complaints Regarding Services Provided by Students
	Inflexibility in Admission Days and Hours
	Long Waiting Time for Services
	Low Durability of Treatments Performed
	Lack of Professional Staff-Patient Interaction
	Insufficient Education, Incomplete Post-Treatment Recommendations and Guidance
	Prolonged Treatment Process due to Training During Treatment
	Dissatisfaction with Treatment Outcome
	Dissatisfaction with Materials Used
	Equipment Shortage
	Imposing Additional Costs on Patients
	Discriminatory Service Provision
	Non-Adherence to Patient Information Confidentiality and Privacy Principles During Service Delivery
	Deficiency in the Service Package Offered
	Lack of Specialist Provider
	Insufficient Faculty Supervision of Student Performance
	Lack of Service Leveling and Referral System
Administrative Bureaucracy	Complexity of Patient Information Dissemination
	Prolonged Patient Introduction Stages by One of the Project Executors
	Patients’ Incomplete Familiarity with Processes
	Deficiencies in Administrative Processes
Lack of Motivation of Stakeholders	Disengagement of Project Executors
	Disheartened Executors Due to Lack of Recognition in Project Launch
	Student Disengagement
	Staff Disengagement
Lack of Institutionalized Inter-sectoral Cooperation	Absence of Permanent Cooperation Between Public Forces and Governmental Institutions
	Claims of Different Groups Regarding Project Ownership and Conflict in Intellectual Property
	Different Perspectives Regarding Project Attribution to Various Organizations
Unsustainable Funding	Lack of Continuity of the IKRF’s Synergy Project
	University Budget Constraints
	Exclusive Cooperation with One Benefactor
	Difficulty in Attracting New Benefactors
	Limited Financial Participation of Patients (No Franchise Fee)
	Inability to Directly Deposit Funds to the Faculty (Service Provider)
Decreased Financial Transparency	Lack of Accurate Auditing
	Decreased Benefactor Trust in Financial Process Integrity
Insufficient Upstream Support	Lack of a Supervisory and Supportive System from the University
	Absence of a Special Supervisor for the Project
Unrealistic Patient Expectations	Incorrect Patient Expectations Regarding Services Provided
	Underestimation of Free Treatment by Patients
Non-Adherence to Social Work Principles	Non-Establishment of a Social Worker in the Faculty
	Provision of Services to Non-Needy Patients and Resource Waste
	Faculty’s Lack of Information on Patient Financial History
Lack of Continuity in service delivery	Incomplete Time Accessibility
	Patient non-commitment to Given Appointments
	Relatively Limited Spatial Accessibility
Lack of Service Support	Lack of Support for Incomplete Services or Treatment Failures
	Failure to Address Complaints
	Staff Disengagement

### Organization of themes using the walt and gilson policy triangle framework

The themes identified in this study were systematically organized according to the four components of the Walt and Gilson policy triangle framework: context, content, process, and actors. This approach provided a structured lens for interpreting the qualitative findings and situating them within broader policy analysis. Each theme was mapped to one of these four categories to ensure a comprehensive understanding of the policy dimensions involved.

## Context

The underlying factors affecting the suspension of the project included management instability, lack of institutionalized inter-sectoral cooperation, and lack of upstream support.

### Management instability

Seven key stakeholders highlighted that the short tenure of the dean of the dentistry school, as the service-providing unit, was a significant factor in the suspension of the project. Frequent managerial turnover in Iran’s public sector often leads to inconsistent decision-making and discretionary actions.

*“*…*Honestly. I’m not sure what happened once the dean got switched. Everything just kind of stopped*,* even though we’d already done all the work!” (Participant 2*,* Faculty Stakeholder).*

Another sub-theme derived from an informed stakeholder’s statements was the “short managerial tenure of the head of the IKRF,” one of the project's key implementing bodies.

*“One of the biggest challenges we ran into was when the head of the IKRF changed*,* and all the issues that came up after that made it hard to keep the projects going” (Participant 12*,* IKRF Stakeholder).*

### Lack of institutionalized inter-sectoral cooperation

One participant mentioned the lack of sustained collaboration between non-governmental organizations, such as charities, and governmental institutions, including universities and the IKRF, as a critical contextual factor influencing both the initial implementation and the mid-term suspension of the project.

“*We don’t really have continuous collaboration in our country where both popular forces and the government manage things together and somehow share leadership” (Participant 7*,* Volunteer Group Stakeholder).*

During the project’s implementation, various stakeholders experienced conflict and tension over the intellectual property ownership of the initiative. This conflict was exacerbated by different ideological perspectives between some faculty members and students.

*“Each group wanted to claim the ownership of the project idea for themselves and tell others*,* look*,* we’re the ones who started and pulled off this great work! No one really cared about cross-sector collaboration or working together to create something genuinely beneficial for the community” (Participant 8*,* Volunteer Group Stakeholder).*

### Insufficient upstream support

The absence of a supervisory and supportive system from the university resulted in the project lacking formal recognition and prioritization by the university president and officials. Consequently, its implementation challenges were neither prioritized for resolution nor supported by the university, leading faculty officials to feel no responsibility or pressure to address the issues, ultimately suspending the project.

*“It’d be better if the university got involved in this project*,*’ cause having the university back the work can really boost its credibility” (Participant 3*,* Faculty Stakeholder).*

*“There should have been an independent person from the university in charge of overseeing the project*,* regularly getting progress reports from the faculty*,* and addressing any complaints if they came up.” (Participant 5*,* Faculty Stakeholder).*

## Content

It consists of four sections: lack of an independent and transparent structure, unsustainable funding, non-adherence to social work principles, and lack of continuity in service delivery.

### Lack of an independent and transparent structure

A key issue with the project was its lack of independent structure. This theme consists of 12 sub-themes. First, ideally, a dedicated office within the faculty should have been established from the outset, with an individual outside the faculty’s academic and administrative staff appointed by the university to oversee the project’s execution from start to finish.

*“Honestly, it would have been better if the project had its own independent office in the dental school from the beginning, and if someone from outside the school had been appointed to manage it (Participant 1, faculty Stakeholder).”*

Furthermore, one of the factors contributing to the project's operational inefficiency was the lack of regular evaluation meetings and the failure to establish a quality management system. It can be inferred that the project suffered from the lack of an appropriate implementation framework, as stakeholders did not study or adopt successful models from similar initiatives. This absence of benchmarking left the project directionless and unprepared to address challenges. Additionally, the lack of long-term planning further undermined the sustainability of the project.

*“This project*,* to me*,* needed a bunch of regular meetings and planning sessions to keep evaluating it and spotting any flaws while it was being carried out” (Participant 3*,* Faculty Stakeholder).*

*“We didn’t even go check out what other groups that pulled off something like this did to start and steer their projects so we could get some help from them” (Participant 3*,* Faculty Stakeholder).*

Three participants mentioned the absence of a vision for potential challenges, which hindered proactive problem-solving and contributed to the suspension of the project.

*“It felt like the people running the project had no idea what challenges might arise along the way*,* or whether they could actually handle them. There didn’t seem to be any clear plan for dealing with potential problems” (Participant 1*,* Faculty Stakeholder).*

Another sub-theme derived from the codes was the “lack of clear and precise role definitions”. Ambiguity in defining roles led to significant operational challenges. For instance, excessive interference by the health benefactor representative in the daily affairs of the dental faculty clinic and the absence of an independent accountant disrupted accountability and workflow, leading to confusion and inefficiency in the project.

Three patients noted that the “irregular and undisciplined attendance of service providers” contributed to “reduced organizational discipline” as a sub-theme, stemming from the inconsistent presence of clinic staff. This lack of consistency in attendance disrupted service delivery and undermined stakeholders’ trust.

Another significant sub-theme highlighted by key stakeholders was the “lack of coordination in supplying consumable materials”. Inadequate initial assessment of material needs and irregular supply processes led to situations where certain materials were depleted prematurely. As students providing services were unable to use the faculty’s materials, this resulted in incomplete patient treatments, causing disruptions in students’ work and dissatisfaction among patients.

*“Because there wasn’t a proper needs assessment for the number of supplies required*,* some materials would run out earlier than expected. And since we weren’t allowed to use the school’s supplies*,* this led to disorganization and sometimes patients’ treatments had to be left unfinished until the following weeks” (Participant 8*,* Volunteer Group Stakeholder).*

Additionally, the procurement of materials by the health benefactor or the IKRF, often involving unapproved brands, occasionally led to dissatisfaction among faculty members and reduced treatment quality for patients. Although two separate cabinets were designated in the dental faculty clinic to distinguish materials purchased for the project from those used for regular faculty operations, staff errors sometimes led to the misuse of these materials. This mismanagement of allocated resources caused an overlap between the faculty’s routine operations and the project, premature depletion of charity materials, and subsequent challenges previously mentioned.

*“One of the problems was that the supplies for the Ghasedak project had to be kept separate from the dental school’s supplies. Even though separate cabinets had been provided*,* sometimes a nurse might unintentionally use the wrong materials*,* which created operational issues” (Participant 2*,* Faculty Stakeholder).*

The project suffered from the simultaneous absence of a separate financial account from the university’s accounts and a lack of cohesive legal foundations. The integration of the project’s financial account into the university’s accounts complicated financial processes due to administrative bureaucracy, leading to delays in transferring health benefactor funds to the faculty for material procurement, causing dissatisfaction and frustration among the health benefactor and the IKRF.

*“Some colleagues were afraid to sign the checks for buying supplies*,* worrying it might cause problems for them later at the university and that they would have to answer for it. The constant managerial changes also meant it took a long time to open a dedicated bank account for the project. All these delays and the unclear operational framework left both the health donor and the IKRF feeling frustrated and discouraged about continuing the collaboration” (Participant 4*,* Faculty Stakeholder).*

Furthermore, the absence of a clear statute and legal framework created operational ambiguity and apprehension among stakeholders regarding their involvement in the project’s implementation.

*“There was no clearly defined job description for either side*,* it wasn’t specified what the school was responsible for or what the IKRF’s obligations were. Also*,* since there was no independent accountant*,* the expenses weren’t properly audited. If there had been a more thorough audit process and the financial reports had been shared between the two parties*,* the whole process would have been more transparent and probably would have led to fewer challenges” (Participant 5*,* Faculty Stakeholder).*

### Unsustainable funding

The lack of continuity in the IKRF synergy program, the university’s limited resources, and the inability to cooperate in procuring consumable materials, prevented the continuation of the project.

*“Back then*,* the IKRF had this idea called the synergy program*,* where they’d say*,* ‘You cover half the cost*,* we’ll cover the other half*,*’ and that teamwork would make things run smoother. So*,* instead of a donor giving forty million*,* he’d give twenty. That plan was a pretty cool incentive for the committee to jump in*,* but sadly*,* that rule’s been scrapped now*,* and the collaborations have gotten worse” (Participant 11*,* IKRF Stakeholder).*

The project’s dependence on a specific benefactor increased its financial and operational risk. Stakeholders stated that difficulties in attracting new benefactors, due to a lack of recognition of dental services as charitable work, hindered the expansion and diversification of the project’s financial resources.

*“Many health philanthropists in the community don’t really see projects like this as real charity. They’d rather fund a hospital or buy a specific piece of equipment*,* which makes it really hard and sometimes frustrating to get them involved in these kinds of initiatives” (Participant 4*,* Faculty Stakeholder).*

Another concern raised by participants was the absence of a franchise fee collection from patients. Collecting a franchise fee would not only enhance the perceived value of the services provided and encourage patients to show greater respect for scheduled appointments and their treatment processes but also contribute to the project’s financial resources for purchasing more and higher-quality consumable materials. This, in turn, would increase patient satisfaction and support the continuation of the project.

*“In our culture*,* free treatment in a lot of cases has kinda shown it doesn’t always carry the value or respect it should*,* and maybe it would’ve been better if we’d charged patients a small deductible” (Participant 8*,* Volunteer Group Stakeholder).*

As previously mentioned under them, lack of an independent and transparent structure, the absence of a separate financial account for the project within the faculty created significant challenges. In this regard, the inability to deposit funds directly into the faculty’s account and the delayed arrival of funds for the scheme’s operational expenses further exacerbated the financial and implementation difficulties of the project.

### Non-adherence to social work principles

The implementation of the project relied entirely on patients referred by the IKRF. However, stakeholders indicated that some referred patients were not genuinely in need. The absence of a social worker within the faculty prevented the faculty from participating in the identification and selection of patients, resulting in services being provided to individuals included on the committee’s list based on relationships rather than eligibility criteria. Establishing a social work unit within the faculty could have facilitated access to patients’ financial backgrounds, ensured the identification of genuinely needy individuals, and prevented potential misuse. This would have promoted equitable service allocation and prevented waste of resources.

*“Some students felt really frustrated when they saw patients coming in with iPhones or wearing lots of gold bracelets*,* clearly well-off*,* yet still receiving free treatment. Meanwhile*,* there are other patients who are genuinely in need*,* but don’t have the money*,* and miss out on care. That’s why the students kept suggesting that*,* besides IKRF patients*,* there should be other ways to identify the people who really need this help” (Participant 17*,* student).*

### Lack of continuity in service delivery

One of the main operational challenges of the project was its restriction to a single day per week (Wednesdays) for patient services. Patients’ lack of commitment to scheduled appointments resulted in some weeks with no patient attendance, leaving faculty members and students without patients and wasting their time. Additionally, the dental clinic’s in-person-only appointment system led to patients outside the project to visit the faculty on Wednesdays without prior knowledge, only to be turned away, causing frustration and wasting their time and resources. Allocating one day per week to the charity prolonged patients’ treatment processes, prevented regular patients from being admitted on Wednesdays even when the faculty was empty, and restricted project patients from receiving services on other days due to concerns about mixing charity and faculty consumable materials. Collectively, these issues created operational challenges for the faculty and led to patient dissatisfaction. Several patients expressed frustration in interviews, noting that the faculty’s distance from their residences and the requirement to attend only on Wednesday mornings made it difficult to take time off work weekly, resulting in their inability to continue their treatment.

*“We had a specific day set aside for the Ghasedak Charity*,* and I think it was Wednesdays when only charity patients were supposed to come in and no outside patients were accepted. This caused problems for us because the number of patients on that day could suddenly be very high or*,* at times*,* very low” (Participant 8*,* Volunteer Group Stakeholder).*

*“There was only one specific day when these services were provided to the public*,* and on other days this option wasn’t available. I went at the beginning of the week*,* but they told me the services weren’t being offered. The dental school was also a bit far away*,* located in the suburbs of Karaj*,* and it was difficult to get there” (Patient 9).*

## Process

### Administrative bureaucracy

Findings showed that “administrative bureaucracy” significantly impacted the implementation phases of project, leading to various challenges.

The coordination process between the IKRF and the faculty including patient referrals, appointment scheduling, and subsequent communication with patients by IKRF was lengthy and complex. This inefficiency resulted in resources wastage and frustration among both patients and providers.

*“For example*,* when we wanted to coordinate*,* there was a long process just to get people scheduled. We’d call and ask them to send a certain number of patients*,* but I’m not sure how the IKRF planned things- sometimes they notified people late*,* and some didn’t show up at all. For instance*,* we might have a list of fifty people waiting to come*,* and only twenty or even ten would actually show up” (Participant 2*,* Faculty Stakeholder).*

One of the primary obstacles to collaboration between governmental and non-governmental organizations lies in their different operational frameworks. To ensure the sustainability and expansion of partnerships between non-governmental organizations and governmental institutions, as observed in the project, administrative processes must be redefined and reformed.

“*Buying stuff in government systems like universities follows some strict bureaucratic rules-purchases must be logged in the electronic procurement system with official invoices. But benefactors often prefer to purchase from a brand offering a higher discount or one that does not require an official invoice. Therefore*,* operational structures are important and need to be redefined” (Participant 5*,* Faculty Stakeholder).*

Another identified subcategory within the category of administrative bureaucracy was the lack of effective communication of procedures to patients and faculty staff. The complexity of notification and correspondence processes led to incomplete coordination among various faculty units, resulting in confusion and inefficiency.

*“One of the problems with this project was that the faculty staff didn’t really know the operational details and weren’t coordinating with each other. It felt like this lack of awareness came from the inefficiency of communication and coordination in our offices*,* which made everything more confusing and frustrating” (Participant 2*,* Faculty Stakeholder).*

### Decreased financial transparency

The complexity of administrative processes, the absence of a charter, the lack of a separate bank account, and the lack of rigorous auditing led to decreased financial transparency, diminished trust from benefactors in the integrity of financial processes, and ultimately their termination of cooperation.

*“The benefactor who was paying for the costs wasn’t sure his money was being used right. He kept saying there should be someone on the faculty overseeing every single step*,* from start to finish*,* to ensure the budget was being spent properly. Honestly*,* from my perspective back then*,* I kept feeling like that was exactly why the project got stopped” (Participant 1*,* Faculty Stakeholder).*

### Lack of service support

The category of “lack of support for services” comprises two subcategories: inadequate support for incomplete or failed treatments and failure to address complaints. One patient, in a tone of frustration and dissatisfaction, stated during an interview, *“The professor told the student who worked on my daughter’s tooth*,* ‘You messed it up*,* and they didn’t even fix it after.’ My daughter’s tooth*,* I think*,* ended up worse*,* and we had to go out and pay for it again” (Patient 11).*

This indicated a lack of follow-up on patients’ conditions post-treatment and a failure by the dental team to rectify treatment errors, leading to poor treatment quality and patient dissatisfaction. Three patients reported that after experiencing complications from their treatments, they returned to the faculty but received inadequate responses from staff and students.

*“After my tooth got infected*,* I went to the dental school and complained*,* asking them to do something. But they didn’t give me a proper response*,* it felt like they were just brushing me off and didn’t really care” (Patient 5).*

## Actors

### Low patient satisfaction stemming from mistrust

Although most patients reported no problems and expressed satisfaction with the care provided by students, a small number offered a contrasting perspective, stating: 

*“Personally*,* I felt really uncomfortable knowing that a new student was going to practice on my teeth” (Patient 9).*

Several patients were dissatisfied with the quality of treatment provided by students. Three patients specifically voiced concerns about the students’ limited experience and skills, which led to prolonged treatment sessions and wasted time.

*“Because the student was too inexperienced*,* it took several sessions just to take the impression of my teeth*,* and honestly*,* it was really frustrating and exhausting for me” (Patient 6).*

Although the majority of patients reported satisfaction with the behavior of the students and faculty staff, one patient expressed a contrasting view, one patient expressed a different opinion and stating that a student yelled at her.

Furthermore, one patient noted feeling uncomfortable and claustrophobic due to many students gathering around her during treatment.

*“There were five or six students around me*,* but they couldn’t extract my tooth properly*,* they were trying too hard that I actually fainted” (Patient 5).*

### Unrealistic patient expectations

The lack of proper patient communication and the provision of inaccurate information led to unrealistic patient expectations regarding the services provided.

*“They told me*,* ‘Go*,* your stuff will be done in a day.’ But that day*,* they just took an X-ray of my teeth and said*,* ‘Come back next week.’ I couldn’t take more time off to go back again” (Patient 22).*

### Lack of motivation of stakeholders

The Safiran labkhand volunteers became discouraged from continuing their voluntary efforts due to a lack of attention and support from senior officials, proprietary attitudes of officials and faculty toward the projects, and the resulting tensions among them. Following the project’s suspension, they did not pursue its resumption. The challenges caused by dedicating only one day per week to the scheme, coupled with patients’ failure to attend scheduled appointments, which left the faculty empty, further demotivated students providing services. From one participant's point of view, the lack of follow-up and seriousness of the students was the main reason for the project’s suspension. One of the participants, a faculty staff member and key implementer of the scheme, expressed frustration, stating that the project’s suspension and the fruitless efforts at its outset led to demotivation and reluctance to participate in similar initiatives in the future. Additionally, the complexity of financial processes for procuring materials, driven by administrative bureaucracy, discouraged the benefactor from continuing their involvement in the project.

*“This project didn’t really have any personal benefit for me; I just wanted to be helpful and feel like I was making a difference for people. If the project had continued*,* and they had asked for my help again on another project*,* I would have participated. But when I saw that it was always the same-that they start something but don’t follow through-I became hesitant. Now*,* if someone else asks me to help with a similar project*,* I think*,* how do I know this won’t turn out like the previous one?” (Participant 1*,* Faculty Stakeholder).*

**Fig. 3:**
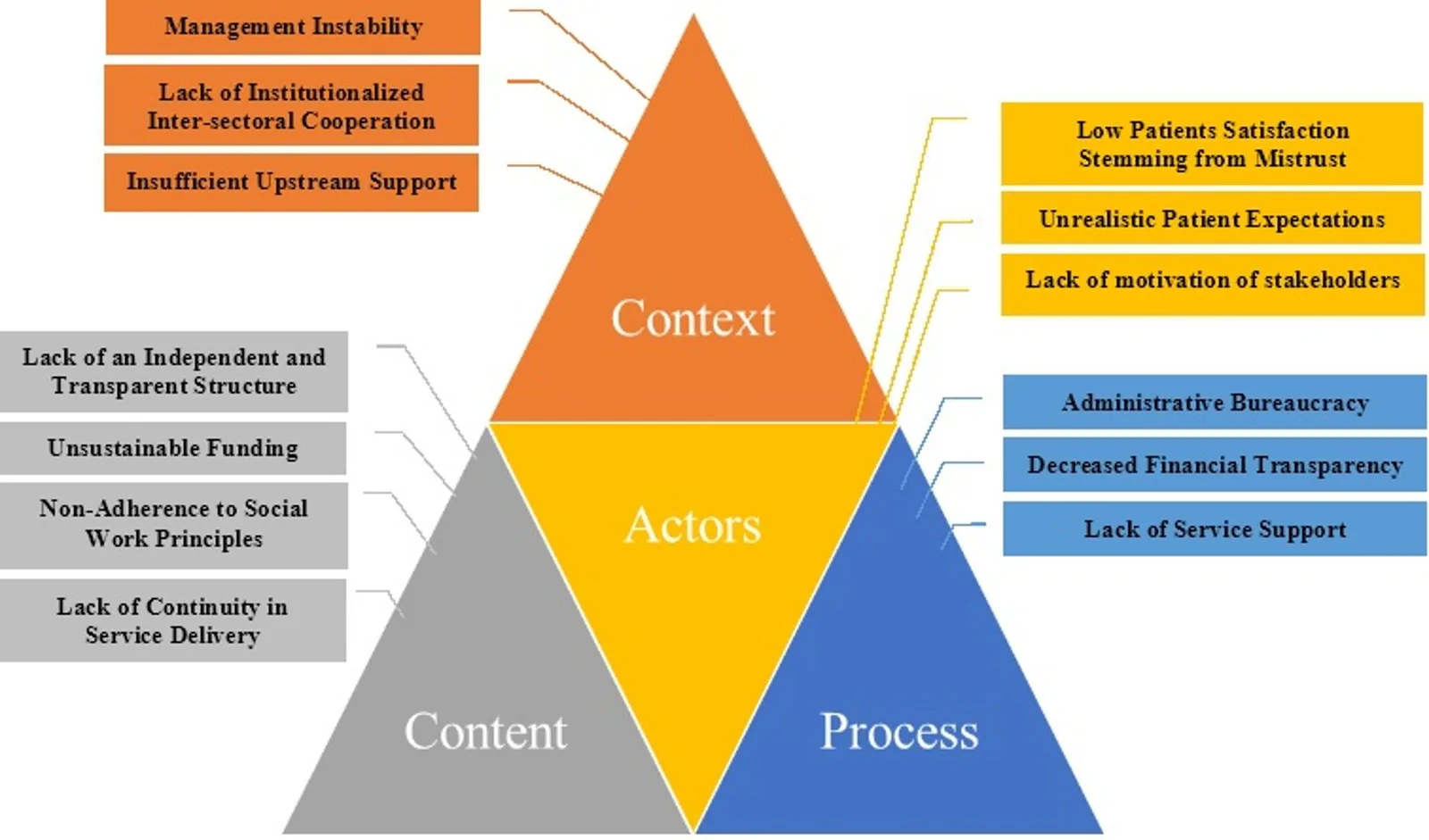
The distribution of themes across the Walt & Gilson framework’s categories

The distribution of themes across the framework’s categories, along with their interrelationships, is illustrated in Fig. 3. This figure shows a visual representation of how the study’s findings align with the triangle policy model and supports clearer interpretation of the results.

## Discussion

Charitable dental initiatives are essential to the dental profession’s ethical commitment to serve those in need. Programs range from those organized by national and state dental associations to faith-based and collaborative efforts with other health professions [25, 26]. Non-governmental organizations (NGOs) in the healthcare sector often face multifaceted challenges that challenge their effectiveness and sustainability. Financial instability [27], poor collaboration with governments, a lack of trust, unclear roles [28], and operational shortcomings, such as inadequate management, human resource limitations, and overlapping services [29], could also limit NGO functions.

The findings indicate a convergence of problems, including acute managerial instability, lack of independent operational and financial structures, unstable funding mechanisms, and discontinuity in inter-organizational cooperation and trust. These challenges are endemic to the non-profit and community health sectors, where high rates of project suspension rates often stem from poor implementation science. Many dental partnerships, for example, fail due to misaligned values and a lack of clear agreements, issues mirrored in the project’s “conflict over intellectual ownership”. Similarly, healthcare public-private partnerships (PPPs) frequently struggle with conflicting goals, imbalanced influence, and a lack of transparency; this parallels the Project experience, where philanthropist trust is reduced due to poor financial transparency.

The financial vulnerability observed in the Project resonates with broader international findings on community dental clinics. Wallace et al. note that while charitable and volunteer-based models are crucial for enhancing access to care, their long-term sustainability is frequently compromised by a reliance on intermittent funding and volunteer goodwill, which cannot substitute for secure structural financing [30]. This fragility suggests that charitable initiatives operating in isolation are prone to implementation challenges. Instead, sustainable reform requires integrating these projects into broader financing mechanisms, similar to strategies proposed for African regions that advocate for combining donor support with health insurance schemes to reduce inequalities [31]. Furthermore, European initiatives like the PRUDENT (Prioritization, incentives and Resource use for sustainable Dentistry) project emphasize that optimizing financing strategies is essential to ensure access without financial hardship, highlighting that good intentions must be backed by evidence-based resource allocation to survive [32].

The actors in the Project were characterized by a duality of disengagement. Patients revealed “low satisfaction and distrust” due to student inexperience, poor communication, and prolonged treatment times. In contrast, key stakeholders, including students and volunteers, suffered a “lack of motivation” due to insufficient support and recognition. While actor dissatisfaction was prominent, the analysis suggests it indicate larger, systemic mistakes. Patient displeasure, for instance, resulted directly from an inefficient service delivery model (a content suspension), while volunteer demotivation was a clear consequence of unstable leadership and a lack of institutional support (a context suspension). While the finding that some patients had “unrealistic expectations” is a common issue in healthcare, it often stems from poor communication and unmet expectations, which are strongly linked to lower satisfaction and loyalty [33].

The project operated in a volatile context, marked by the “short managerial tenure” of key leaders, a “lack of institutionalized inter-sectoral cooperation,” and an absence of “oversight from higher authorities”. Managerial instability disrupts funding, erodes morale, and leads to a loss of institutional knowledge [34, 35]. The project’s stoppage after a change in leadership is a classic example of this weakness. In addition, the lack of institutionalized cooperation and ownership disputes are common downsides in healthcare partnerships, which require trust and a shared vision to succeed [36]. The project existed in a policy and governance vacuum and was not institutionalized as a core function of its parent organizations. Without this institutional scaffolding, it was highly susceptible to external shocks and internal friction that a formally integrated program could better tolerate. Moreover, the Project’s isolation contrasts with integrated care models like the Community Oral Health Transformation initiative in North Carolina[37], which emphasizes medical-dental integration and value-based care to advance health equity. These examples suggest that for the Iranian dental payment initiative to succeed, it must move beyond simple service provision and adopt a community-driven and structurally integrated approach.

The project’s content was fundamentally weak, suffering from a “lack of independent structure,” “unstable financial provision,” and “non-adherence to social work principles”. The absence of a strategic and action plan or quality management system contrasts sharply with best practices for non-profit management [38]. The financial model, relying on a single benefactor and lacking patient co-payment, leads to poor financial management, creating extreme susceptibility [39, 40]. The entire content of the project was tactical and short-term, designed for initiation rather than sustainability, with little strategic thought given to the structures needed to sustain it over time.

The project’s processes were overwhelmed by “administrative bureaucracy,” “decreased financial transparency,” and a “lack of service support”. These implementation challenges are common in large-scale healthcare projects where inadequate planning and poor communication lead to discontinuation [41, 42]. The loss of philanthropist trust due to a lack of financial transparency was a critical challenge in implementing the process that severed the project’s financial lifeline; transparency is principal for building and maintaining donor trust [42]. The project was caught in a bureaucratic trap, hindered by incompatibilities between the incompatible administrative systems of its partner organizations, which is a key reason interorganizational collaborations fail [43, 44].

In synthesizing the findings of this study using the Walt and Gilson policy triangle, the failure of the program can be understood as the result of interacting weaknesses across all four dimensions of the framework. At the level of content, the absence of comprehensive pre‑implementation planning and the lack of a transparent structural design undermined the program’s coherence and operational clarity. Regarding process, insufficient anticipation of organizational culture challenges and limited assessment of long‑term service delivery capacity compromised implementation sustainability. In terms of context, inadequate institutional backing from higher-level authorities, combined with frequent managerial turnover, created instability and weakened policy continuity. Beyond these structural and managerial shortcomings, a critical insight emerging from this case concerns the actors dimension. A misalignment between patients’ needs and the program’s eligibility criteria and operational requirements contributed to dissatisfaction, erosion of trust, and, in some instances, inefficient use of charitable resources. This finding underscores the importance of early-stage stakeholder engagement and transparent communication with service recipients in charity-based health initiatives.

Conceptually, this case illustrates that in charity-driven dental programs, the actors dimension may play a mediating role that amplifies weaknesses in content, process, and context. In settings such as Iran, where strong cultural norms of altruism facilitate the frequent implementation of short-term charitable healthcare initiatives, programs are often designed as brief, cross-sectional interventions that can operate with limited structural complexity. However, long-term initiatives, such as the Ghasedak project, require a fundamentally different policy architecture: one grounded in sustainable financing, stable governance structures, and continuous alignment between program design and beneficiary needs.

Therefore, this study extends the application of the Walt and Gilson framework by demonstrating that the sustainability of dental payment or service reforms in charity-based contexts depends not only on balanced attention to the four policy dimensions, but also on the dynamic alignment between beneficiary expectations and institutional capacity. This conceptual refinement moves beyond descriptive analysis to offer a more nuanced understanding of policy vulnerability in long-term oral health initiatives.

### Strengths and limitations

This study possesses several methodological strengths. First, its application of the Walt and Gilson health policy triangle moves the analysis beyond simple description to a theoretically grounded framework, enhancing the rigor and transferability of the findings. Second, 360-degree stakeholder participation, involving 47 interviews with a comprehensive range of participants, provides a rich and holistic view that strengthens the validity of the conclusions. Third, the qualitative methodology enabled an in-depth exploration of the “how” and “why” behind the project’s challenges, capturing nuances that quantitative data cannot capture. Finally, the systematic analytic process, progressing from 145 open codes to higher-order categories prior to framework mapping, enhanced the rigor and transparency of interpretation. Given that this case is situated within a government dental education setting, which remains underrepresented in the literature, the findings are not intended to be statistically generalizable; rather, they offer analytical and conceptual transferability to comparable public dental school and training contexts in low- and lower-middle-income countries (LMICs), including Iran. This study is not without limitations. Its findings have limited generalizability, though the conceptual insights are transferable as a single case study. The retrospective design introduces potential selection bias due to non-random recruitment, social desirability bias, and recall bias, as participants’ memories may be imperfect or colored by the project’s adverse outcome. Despite the research team’s efforts to purposively sample both women and men, the high volume of service recipients and the much higher response rate among women resulted in a predominantly female sample of patients (26 women vs. 2 men), which may have led to sampling bias.

### Recommendations

- Establish clear legal frameworks and semi-autonomous governance structures to protect charitable dental initiatives from bureaucratic instability.
- Design flexible, multi-day service schedules and integrated appointment systems to ensure continuity of care and improve patient retention.
- Implement transparent communication and shared oversight mechanisms to sustain trust among institutional and philanthropic partners.
- Adopt co-design approaches involving insurers, academic leaders, providers, and patients from the early planning stages.
- Create legally autonomous and transparent funding mechanisms to simplify financial processes and encourage long-term benefactor engagement.
- Ensure managerial stability and clear operational authority to improve coordination, accountability, and program effectiveness.

## Conclusions

This qualitative policy analysis indicates that the pilot initiative encountered multiple, interrelated challenges arising from contextual instability, design limitations, administrative complexity, and variable stakeholder engagement. The findings, rather than a single implementation challenge, highlight how misalignment across policy content, governance arrangements, and implementation processes can undermine the sustainability of community oral health initiatives.

The study suggests that the effectiveness of similar programs may depend on closer integration of policy objectives with operational capacities, particularly regarding leadership continuity, governance clarity, and financial arrangements. Looking forward, future payment reforms in community oral health could benefit from early engagement of key stakeholders, including policymakers, service providers, and financing bodies, as well as from the articulation of transparent and predictable financial mechanisms. Such considerations may help inform the design and implementation of more resilient and context-sensitive oral health policies.

## Supplementary Information

Supplementary Material 1.

## Data Availability

Data available upon reasonable request from the corresponding author.

## Abbreviations

WHO: World Health Organization
UHC: Universal health coverage
MoHME: Ministry of Health and Medical Education
IKRF: Imam Khomeini Relief Foundation
ABZUMS: Alborz University of Medical Sciences
NGO: Non-governmental organization
PPP: Public-private partnership
