# Supplementary material for: Exploring the challenges of a pilot dental services payment initiative: a qualitative policy analysis at an Iranian dental school
Source: BMC Oral Health. 2026 Jun 29;26:1864. doi: 10.1186/s12903-026-08767-9 (PMC13621463; doi:10.1186/s12903-026-08767-9)
Supplement: Supplementary file 1 [file 12903_2026_8767_MOESM1_ESM.pdf]

Semi-structured interview guide

**1. Participant name**	
**2. Participant ID**	
**3. Interview Date**	
**4. Interview Location**	
**5. Participant agrees for interview to be digitally recorded**	**Yes**..☐<br>**No**...☐
**6. Time Interview Began**	
**7. Time Interview Ended**	

Step 1: Complete Q1-4 above before the interview.

Step 2: At the beginning of the interview, introduce yourself; thank participant for taking part in the interview.

Step 3: Complete demographic information

Step 4: Ask participant permission to record interview; tick appropriate box in Q5 above.

Step 5: Turn on audio recorder if acceptable, document time interview begins in Q6 above, and conduct interview according to section A-D.

Step 6: At the end of the interview, thank the participant and ask if she/he has any further questions; document time interview ended in Q7 above.

Section A: Warm Up

- What was your experience with implementing the Ghasedak Charity project?
- What do you think were the advantages of this project?
- What do you think were the disadvantages of this project?

Section B: Walt and Gilson's main questions

- What do you think were the contexts for implementing the project?
- What do you think was the content of implementing the project?
- What do you think were the processes for implementing the project?
- Who do you think were the stakeholders in implementing the project?
- How did implementing the project affect each stakeholder?

Section C: Conclusion

- if you see any other flaws in this project that haven't been mentioned in the interview so far, please tell me now.

Section D: Recommendation

- What do you think are the solutions for continuing this project?
